# A Case of Fatal Poisoning by Chininum Sulph.

**Published:** 1888-04

**Authors:** 


### A CASE OF FATAL POISONING BY CHININUM SULPH.

(From *Allg. Med. Centr. Zeitung.*)

Two soldiers, thinking they were drinking a solution of Glauber's salts, swallowed each a cupful of a five per cent. solution of Quinine. All the tissues of the body soon turned pale, especially the face; the pupils dilated to their utmost, the eyes became protruded and staring; the breathing was accelerated with dyspnœa; pulse retarded, small, and from time to time filiform, irregular, and intermittent; the temperature of the body diminished. It seemed as though the nervous system were not attacked at all. One died after two hours, the other recovered rapidly. The fatal dose was twelve grammes, whereas it has been asserted that it takes thirty-five grammes to produce dangerous symptoms of poisoning. In Allen's *Encyclopædia* we read: Symptoms—161, disk and retina very anæmic; 168, pupils very much dilated; 170, dimness of sight, even to blindness; 245, face pale and suffering; 250, great paleness of face; 669, acceleration of heart's beat; 720, temperature depressed; 721, pulse regular, very weak, scarcely perceptible; 759, weakness of limbs, they will not obey the will; 1010, general chilliness over the whole body, especially the back; icy coldness of the body.

